# Supplementary material for: Exploring the Putative Involvement of MALAT1 in Mediating the Beneficial Effect of Exendin-4 on Oleic Acid-Induced Lipid Accumulation in HepG2 Cells
Source: Biomedicines. 2025 Feb 5;13(2):370. doi: 10.3390/biomedicines13020370 (PMC11853215; doi:10.3390/biomedicines13020370)
Supplement: Supplementary file 1 [file biomedicines-13-00370-s001.zip › biomedicines-3421462-supplementary.pptx]

## Slide 1
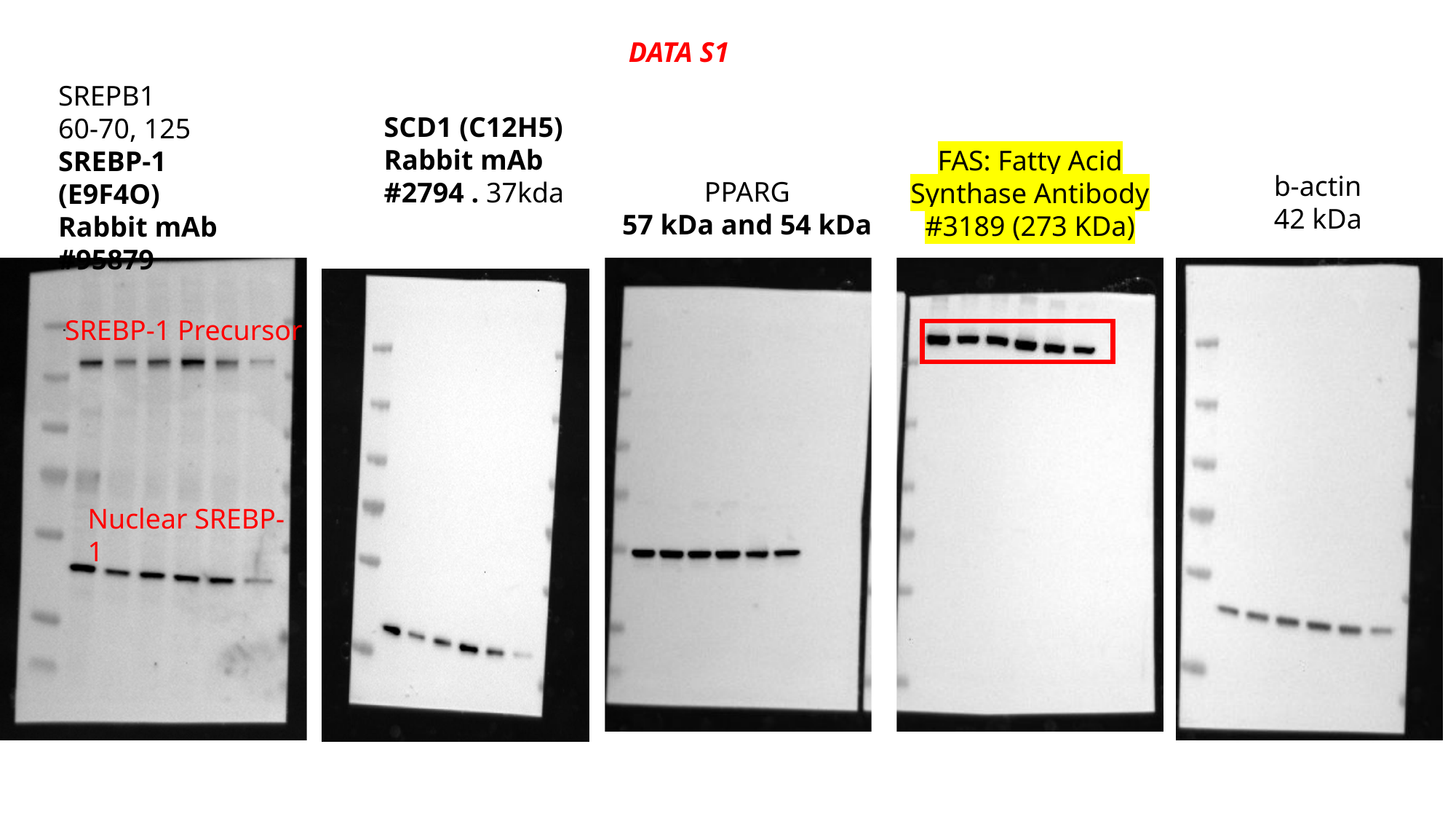

DATA S1
SREPB1
60-70, 125
SREBP-1 (E9F4O) Rabbit mAb #95879
SCD1 (C12H5) Rabbit mAb #2794 . 37kda
FAS: Fatty Acid Synthase Antibody #3189 (273 KDa)
b-actin
42 kDa
PPARG
57 kDa and 54 kDa
SREBP-1 Precursor
Nuclear SREBP-1

## Slide 2
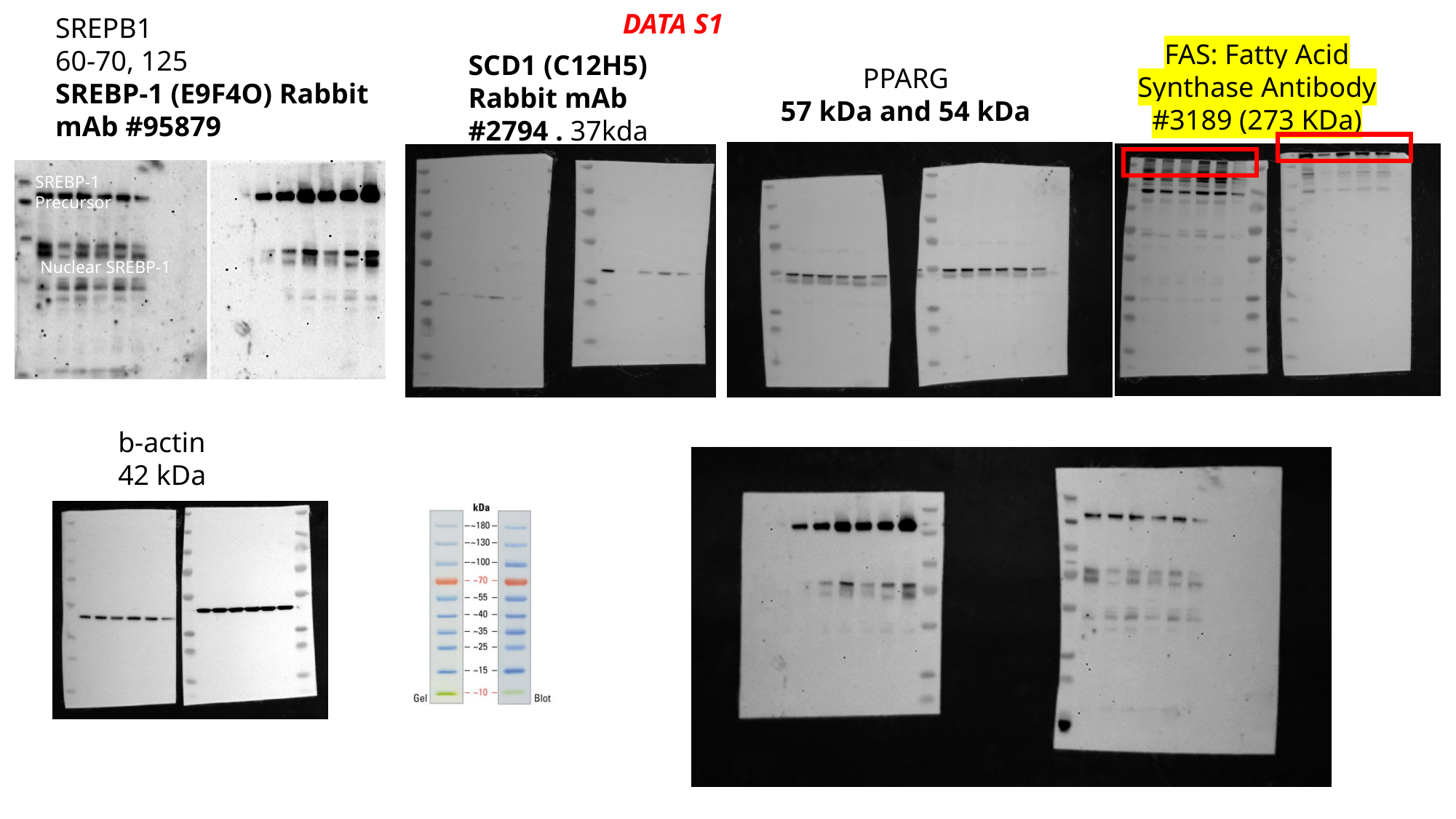

DATA S1
SREPB1
60-70, 125
SREBP-1 (E9F4O) Rabbit mAb #95879
FAS: Fatty Acid Synthase Antibody #3189 (273 KDa)
SCD1 (C12H5) Rabbit mAb #2794 . 37kda
PPARG
57 kDa and 54 kDa
SREBP-1 Precursor
Nuclear SREBP-1
b-actin
42 kDa

## Slide 3
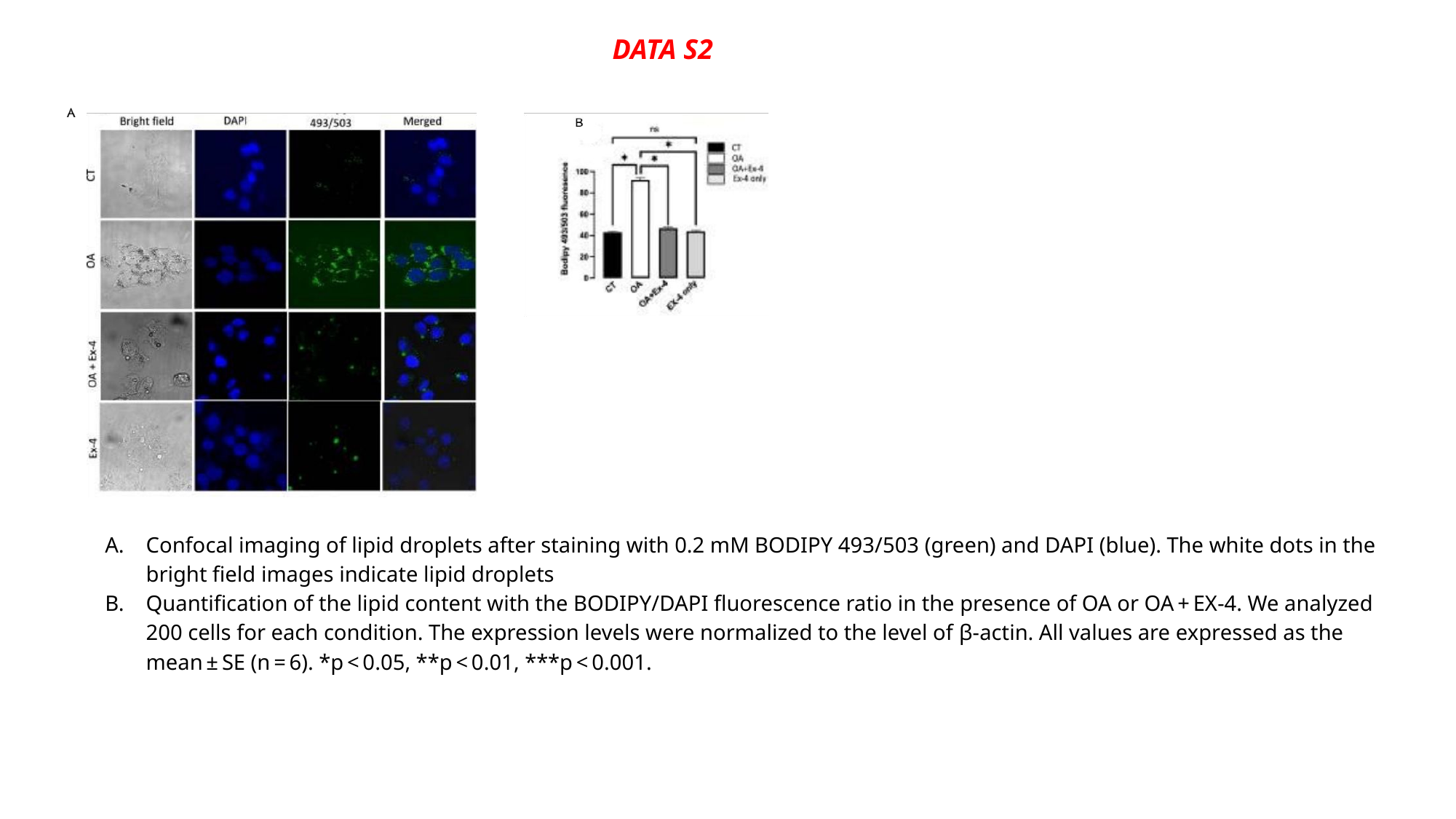

DATA S2
Confocal imaging of lipid droplets after staining with 0.2 mM BODIPY 493/503 (green) and DAPI (blue). The white dots in the bright field images indicate lipid droplets
Quantification of the lipid content with the BODIPY/DAPI fluorescence ratio in the presence of OA or OA + EX-4. We analyzed 200 cells for each condition. The expression levels were normalized to the level of β-actin. All values are expressed as the mean ± SE (n = 6). *p < 0.05, **p < 0.01, ***p < 0.001.
